# Mitochondrial quality control and neurodegenerative diseases

**DOI:** 10.1042/NS20180062

**Published:** 2018-12-03

**Authors:** Fei Gao, Jianmin Zhang

**Affiliations:** Department of Immunology, Research Center on Pediatric Development and Diseases, Institute of Basic Medical Sciences, Chinese Academy of Medical Sciences and School of Basic Medicine, Peking Union Medical College, State Key Laboratory of Medical Molecular Biology, Beijing 100005, China

**Keywords:** mitochondria, neurodegeneration, quality control

## Abstract

Mitochondria homeostasis is sustained by the mitochondrial quality control (MQC) system, which is crucial for cellular health, especially in the maintenance of functional mitochondria. A healthy mitochondria network is essential for life as it regulates cellular metabolism processes, particularly ATP production. Mitochondrial dynamics and mitophagy are two highly integrated processes in MQC system that determines whether damaged mitochondria will be repaired or degraded. Neurons are highly differentiated cells which demand high energy consumption. Therefore, compromised MQC processes and the accumulation of dysfunctional mitochondria may be the main cause of neuronal death and lead to neurodegeneration. Here, we focus on the inseparable relationship of mitochondria dynamics and mitophagy and how their dysfunction may lead to neurodegenerative diseases.

## Introduction

Mitochondria are semi-autonomous organelles known as the powerhouse of cells, generating ATP via the oxidative phosphorylation (OXPHOS) system. The mitochondrion possesses a dual membrane structure: the outer mitochondrial membrane (OMM) interacts with the cytoplasm, and the inner mitochondrial membrane (IMM) is folded into the cristae and contains the mitochondrial matrix. Mitochondria are vital in controlling calcium homeostasis, generating reactive oxygen species (ROS), modulating signaling transduction, and deciding cell fate [1–3]. These functions are highly dependent on the homeostasis of the mitochondrion network. Therefore, the mitochondrial quality control (MQC) system is important in the maintenance of mitochondria function. Mitochondria experience constant dynamic changes, including fusion and fission, to sustain their integrity and satisfy metabolic demands at the level of the organelle [4]. If damage is beyond repair, the dysfunctional mitochondria are engulfed in double-membrane phagosomes and fused to lysosomes for degradation through mitophagy [5,6]. Neurons have a high demand for energy consumption, and thus damaged mitochondria may lead to neuronal death [7]. Moreover, much evidence has suggested that impaired MQC system contribute to the progression of the pathogenesis of neurodegenerative diseases [8–10]. In this review, we will discuss the mechanisms within the MQC system, their co-operation in the sustaining of a healthy mitochondria network, and how their dysfunction may cause neurodegenerative disorders.

## Cellular and molecular processes of MQC (What is MQC?)

Mitochondria undergo continuous dynamic processes that not only regulate their morphology and size but also decide whether damaged mitochondria are to be repaired or degraded [11]. In the life cycle of mitochondria, fission, fusion, and subcellular translocation always occur, placing the whole mitochondrion network in a constant dynamic process that not only remodels mitochondrial size but also maintains its normal function [12]. Fission leads to uneven generation of daughter mitochondria, where the depolarized mitochondrion loses its normal function and easily undergoes mitophagy [13]. In contrast, fusion results in elongated mitochondria, which contributes to the preservation of crucial proteins for meeting the increased energy and metabolic demand in cells, thus protecting cells against death or autophagy.

Mitochondrial fission is mainly mediated by dynamin-related protein 1 (Drp1), an evolutionarily conserved GTPase in eukaryotic cells that is normally distributed in the cytosol [14,15]. Different cellular processes induce unique signals that cause distinct DRP1 post-modifications; however, the specific mechanisms involved need further investigation [16]. Fission protein 1 (Fis1) and mitochondrial fission factor (Mff) are regarded as major Drp1 receptors, and their reduced expression causes a decline in Drp1 recruitment and results in elongated mitochondria [17]. In contrast, overexpressed Mff leads to an increased number of fragmented mitochondria [18]. Recent research has shown that two homologous proteins, Mid49 and Mid51 (MIEF1), are new mediators that promote the recruitment of Drp1 to OMM [19]. In the process of mitochondria fission, GTP hydrolysis drives self-assembled spiral Drp1 to constrict mitochondrial tubules, thus separating both the IMM and OMM. Evidence shows that the endoplasmic reticulum (ER) interacts with mitochondria before DRP1 recruitment, thereby narrowing mitochondrial tubules and facilitating mitochondria division [20]. After the cycle of mitochondrial fission, Drp1 spirals disassemble for the next mitochondrial fission cycle. However, recent studies have demonstrated that other OMM proteins or even exogenous molecules can also lead to mitochondrial fission in a DRP1-independent way, indicating other potential mechanisms that regulate mitochondrial fission [21,22].

Mitochondrial fusion is a two-step process mediated by OMM protein mitofusins (Mfn1 and Mfn2) and IMM protein optic atrophy 1 (OPA1) [11]. While the mitofusin-mediated mitochondria physical interaction is GTP-independent, GTP hydrolysis is indispensable in the process of OMM fusion. After two mitochondria physically interact with each other, mitofusins insert into the OMM, thus mediating the mixture of OMM structure in either a homotypic or heterotypic way [23]. However, only Mfn2 tethers ER to mitochondria, mediating calcium uptake in mitochondria-ER/sarcoplasmic reticulum and regulating calcium signaling in ER stress [24,25]. OPA1 is located on the IMM and mediates its fusion, whereas Mfn-mediated OMM fusion occurs in a GTP-dependent manner [26]. OPA1 dysfunction not only results in fragmented mitochondria but also disturbs the inner membrane potential, which suggests its role in mediating electron transport chain (ETC) complexes as well as cell fate [27,28]. Furthermore, the functions of these dynamic proteins are not separate. The whole mitochondrial dynamic network is regulated in a complex but delicate system. As will be discussed later, the pathological implications of these mitochondrial dynamic factors extend far beyond simply regulating mitochondrial morphology.

Mitophagy is a highly conserved process in which damaged mitochondria are engulfed in double-membrane autophagosomes and transferred to lysosomes for degradation and recycling [29]. Mitophagy is believed to have a central role in the MQC system. Eukaryotic cells process several different mechanisms inducing mitophagy, of which PINK1-Parkin pathway is the most well-known [30]. When mitochondria depolarization is irreversible, PTEN induced putative kinase 1 (PINK1) is recruited to OMM and phosphorylates its downstream proteins, ubiquitin and Parkin. Parkin ubiquitination activates its E3-ligase activity, thus binding to downstream mitophagy receptors and leading to the formation of LC3-positive autophagosomes that warp the damaged mitochondria [31]. Recent evidence shows that Mfn dysfunction disrupts the mitochondrial membrane potential, preventing Parkin’s translocation from the cytosol to OMM [32]. Moreover, PINK1 or Parkin deletion can both hinder mitophagy and result in elongated mitochondria simultaneously [33].

**Figure 1:**
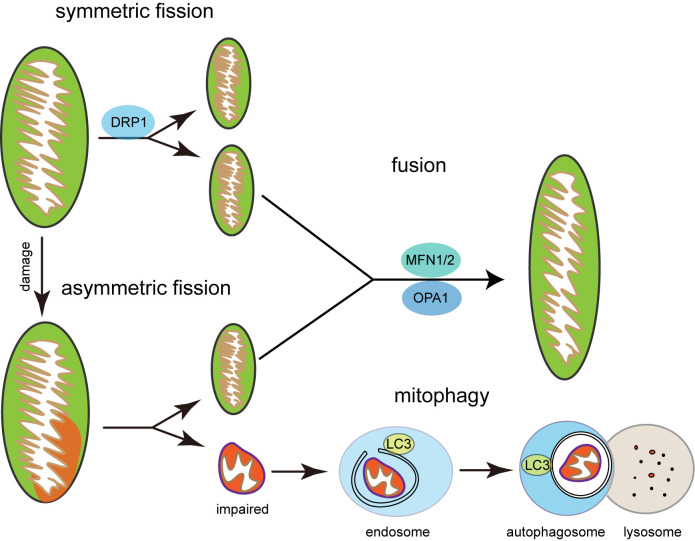
The regulation of MQC system Symmetrical fission results in two healthy daughter mitochondria, while asymmetrical fission leads to selective mitophagy of the impaired mitochondria.

Mitochondria dynamics and mitophagy are highly integrated (Figure 1). Mitochondria experience symmetrical fission, replicating itself into two healthy daughter mitochondria, whereas damaged mitochondria experience asymmetrical fission, which is an economical way of recycling functional parts from the damaged organelle resulting in the biogenesis of one healthy mitochondrion and one depolarized mitochondrion [34]. The depolarized mitochondrion will then undergo the process of mitophagy. A recent study demonstrated that the loss of DRP1 increased mitochondria length and defected PINK1-Parkin-independent mitophagy in mouse brain, resulting in a neurodegenerative phenotype [35]. The study also showed that Mfn2 plays a role as a phosphorylation substrate of PINK1, which is phosphorylated at Thr^111^ and Ser^442^, ensuring binding of Mfn2 and Parkin, thereby suggesting a potential pathway in the MQC process [36]. As demonstrated above, a subtle change in the whole MQC system would affect mitochondria homeostasis greatly.

## MQC and mitochondrion damage (Why is MQC important?)

Mitochondria experience constant tiny but not serious damage. The MQC system is an effective weapon maintaining mitochondrial health. While neuronal mitochondria have a long longevity, it is likely that they suffer from accumulated damage [37], which compromises mitochondrial function, disturbs mitochondrial homeostasis, and eventually causes neuronal death.

Mitochondrial dysfunction occurs under many conditions. Dysfunctional mitochondria experience increased oxidative damage which causes the decline in energy production, reduced enzyme catalytic activity, and changes in protein function. Mitochondria are semi-autonomous organelles that contain their own DNA (mtDNA) [38]. Tons of studies have demonstrated a strong link between mtDNA mutation and mitochondria damage. Point mutations and gene deletion are the two most frequent mtDNA damages in mitochondria. In addition, mtDNA insertion and gene copy number variation also interrupt the stability of the mitochondrial internal environment and lead to neuronal death [39–42]. ROS are generated as natural byproducts of oxidative metabolism and can be rapidly removed by the generation of H_2_O_2_ through the help of superoxide dismutases (SODs). The imbalance between ROS generation and the impaired antioxidant process leads to excessive ROS accumulation, causing peroxidation of biomacromolecules including lipid, proteins, and nucleic acids, which eventually lead to cell death and neurodegeneration [29,43]. The ATP is generated through a combination of the tricarboxylic acid (TCA) cycle and proton transport on ETC at the inner membrane through the OXPHOS system [38,44]. The ETC complex consists of a series of multi-subunit complexes (I, II, III, and IV) that drive the formation of a proton gradient. The perturbation of the normal function of these complexes easily reduces the ATP production, resulting in difficulty in meeting the metabolic needs of the cell [45,46]. In addition, Ca^2+^ retention is also important in the maintenance of mitochondrial membrane potential, the loss of which leads to mitochondria dysfunction and cell apoptosis [47].

The MQC system plays a vital role in the maintenance of mitochondria homeostasis and neuronal health. Therefore, developing measures to enhance mitochondrial function via the MQC system is of great importance for the clearance of damaged mitochondria and for promoting a healthy mitochondrial network.

## MQC deficiency and neurodegenerative diseases

Since mitochondria deficiency and dysfunctional MQC system are regarded as common features in neurodegenerative diseases, herein, we will discuss their role in the pathological progress of several neurodegenerative disorders.

### Alzheimer’s disease

Alzheimer’s disease (AD) is the most common neurodegenerative disease among the elderly, causing a severe economic burden in society [48]. The pathological hallmark that characterizes AD includes extracellular Aβ deposition and intracellular hyperphosphorylated tau containing neurofibrillary tangles [49]. Although the specific mechanism causing AD still remains to be discovered, dysfunctional MQC system that progress AD pathologies have been found. An interesting study showed that while the fusion protein is decreased, fission-associated protein Fis1 is increased in the brain of AD patients, indicating a disrupted mitochondrial dynamic process [50]. A transcriptome and proteome study in AD patients also demonstrated the same result [51]. An *in vitro* experiment found that the fibroblasts of AD patients showed an increased number of elongated mitochondria aggregated around the nucleus that was accompanied by a decreased level of DRP1 [52]. Therefore, extracellular Aβ deposition and mitochondria dysfunction are closely linked. Another study found that damaged mitochondria can easily accelerate Aβ deposition [53]. In contrast, Aβ aggregation leads to excessive ROS accumulation, thus causing mitochondria dysfunction [54]. The deposition of oligomeric Aβ reduced mitochondria number and caused fragmented mitochondria, which could be rescued by overexpression of DRP1 [50]. With the accumulation of damaged mitochondria, a compromised mitophagy process is also an important aspect that leads to AD pathology. A mutation in the AD-related protein presenilin-1 (PS1) elevated lysosomal pH, thereby reducing its hydrolase activity that inhibits the clearance of autophagosomes [55]. Together, these findings demonstrate that defected MQC system are essential in AD pathogenesis.

### Parkinson’s disease

Parkinson’s disease (PD) is the second most common neurodegenerative disease and is characterized by dopamine neuronal loss in the substantia nigra as well as intracellular α-synuclein aggregation [56]. The clinical feature of PD is a progressive movement disorder [57]. In recent years, genome-wide association studies (GWAS) have associated MQC genes and their products with the progression of PD [58,59]. Mutations in either PINK1 or Parkin are the most well-known causes of autosomal recessive forms of PD [60,61]. Studies of mutant PINK1 or Parkin in *Drosophila* models demonstrated that degenerated DA neurons are filled with swollen mitochondria [62–64]. Conditional PINK1 knockout in mouse substantia nigra directly leads to dopamine neuronal death [65]. However, in the cell model of PINK1 knockout mammalian neurons, calcium accumulated in the mitochondria, resulting in increased ROS production, and ultimately cell death [66]. Mutant LRRK2 is associated with autosomal-dominant PD [67]. Under physiological conditions, LRRK2 interacts with mitochondrial dynamic protein, regulating the balance of the mitochondrial network [68,69]. Studies have demonstrated that G2019S mutant LRRK2 cells have a decreased level of ATP production accompanied by mitochondrial uncoupling and a compromised mitophagy process [70,71]. Impaired mitophagy facilitates the deposition of α-synuclein, which aggregates into oligomers and leads to cell dealth [72,73].

### Huntington’s disease

Huntington’s disease (HD) is an inherited neurodegenerative movement disorder that mostly occurs in the middle-aged population [74]. A variation in CAG copy number of the *HTT* gene is regarded as the main reason causing striatal GABAergic neuronal loss. The brain tissue from HD patients exhibits highly fragmented mitochondria as well as impaired respiration function [75]. Mutant Htt is strongly neurotoxic. Both *in vivo* and *in vitro* experiments have demonstrated that mutant Htt disturbs calcium buffering capacity, thereby reducing mitochondrial membrane potential, impairing OXPHOS process, and resulting in mitochondrial damage [76,77]. A shift from fusion to fission is observed in Htt mutant mitochondria [78]. Recent findings demonstrated that DRP1 is a potential target that mutant Htt can bind to disturb the DRP1 assembling process. These findings suggest a strong link between mitochondrion dynamics and HD [79,80].

### Amyotrophic lateral sclerosis disease

Amyotrophic lateral sclerosis disease (ALS) is a late-onset motor neuronal affected neurodegenerative disease [81]. Mutations in TDP-43 and Cu/Zn-binding SOD1 are the main causes of ALS. Mutant SOD1 preferentially binds to mitochondria, thus interfering with the OXPHOS process and resulting in damaged mitochondria as well as disrupted mitochondrial transportation [82,83]. Both TDP-43 overexpression and mutation decreased mitochondrial length and density, which could be reversed by co-expression of Mfn2 [84]. The effective removal of abnormal protein is crucial in maintaining motor neuronal health in ALS [85]. However, the impaired capability of activating effective autophagy is the consequence of ALS [86].

### Charcot–Marie–Tooth neuropathy type 2A

Charcot–Marie–Tooth neuropathy (CMT) type 2A (CMT2A) is a heterogeneous neurodegenerative disorder characterized by peripheral axonal degeneration of long sensory and motor neurones. Although the mechanism of this disease is not well-known, Mfn2-induced mitochondria dynamic dysfunction plays an important role in the pathogenesis [87]. Mfn2 deficiency hinders mitochondria dynamics toward fusion, which results in an inability to meet the metabolic needs of these long sensory motor neurons. Moreover, Mfn2 deficiency reduces ATP production, thus progressing CMT2A pathology [88].

## Concluding remarks

Significant progress has been made in understanding the mechanisms between MQC dysfunction and neurodegenerative diseases. A healthy mitochondrial network is sustained by the co-operation of both mitochondrial dynamics and an effective mitophagy pathway. Investigations into how mitochondria damage occurs in each neurodegenerative disorder will elucidate potential therapeutic targets in each disease. The future goal of our studies is mainly focussed on personalized and accurate modulation of MQC system.

## Abbreviations

AD: Alzheimer’s disease
ALS: amyotrophic lateral sclerosis disease
CMT2A: Charcot–Marie–Tooth neuropathy type 2A
DRP1: dynamin-related protein 1
ER: endoplasmic reticulum
ETC: electron transport chain
Fis1: fission protein 1
HD: Huntington’s disease
IMM: inner mitochondrial membrane
Mff: mitochondrial fission factor
MQC: mitochondrial quality control
OMM: outer mitochondrial membrane
OPA1: optic atrophy 1
OXPHOS: oxidative phosphorylation
PINK1: PTEN induced putative kinase 1
ROS: reactive oxygen species
SOD1: superoxide dismutase 1
